# Comprehensive SSR study of 14 Zingiberaceae species based on microsatellite capture sequencing (MiCAPs)

**DOI:** 10.3389/fpls.2025.1693622

**Published:** 2025-12-11

**Authors:** Miao Shi, Keisuke Tanaka, Marlon P. Rivera, Min San Thein, Godfrey M. Ngure, Kazuo N. Watanabe

**Affiliations:** 1 Degree Programs in Life and Earth Sciences, Graduate School of Science and Technology, University of Tsukuba, Tsukuba, Japan; 2 Department of Informatics, Tokyo University of Information Sciences, Chiba, Japan; 3 Institute of Biological Sciences, University of the Philippines Los Baños, Laguna, Philippines; 4 Department of Agricultural Research, Industrial Crops and Horticulture Division, Republic of the Union of Myanmar, Yezin, Myanmar; 5 Tsukuba-Plant Innovation Research Center, Institute of Life and Environmental Sciences, University of Tsukuba, Tsukuba, Japan

**Keywords:** MICAPS, SSR, microsatellite, *Kaempferia*, *Zingiber*, *Curcuma*, *Hedychium*, *Rhynchanthus*

## Abstract

**Introduction:**

The Zingiberaceae family encompasses numerous species renowned for their significant pharmacological properties and culinary importance. Despite this value, many species remain under-utilized due to the absence of basic molecular information, which hinders effective conservation and sustainable utilization. Simple Sequence Repeat (SSR) markers are particularly suitable for genetic studies in such species, as they are highly polymorphic and do not necessitate a reference genome. Microsatellite capture sequencing (MiCAPs) presents a cost-effective solution by enriching libraries for SSR-containing fragments prior to sequencing, substantially reducing data requirements and costs for marker discovery.

**Methods:**

MiCAPs was applied to 160 accessions, including 148 samples from 14 Zingiberaceae species and 12 samples from an outgroup (Musaceae family). SSR marker candidates were developed and evaluated via electronic-PCR (ePCR) for seven target species. Phylogenetic relationships were reconstructed using consensus sequences from MiCAPs data, and genetic similarity patterns were assessed using Polymorphic SSR Retrieval (PSR) analysis across *Curcuma* and *Zingiber* species.

**Results:**

A total of 21.78 million raw reads were generated, from which 612 SSR marker candidates were developed. A genus-level phylogenetic tree successfully reconstructed the relationships among the 14 Zingiberaceae species. Comparative genetic diversity analysis revealed that *Zingiber* exhibits a relatively more conserved genetic background compared to *Curcuma*.

**Discussion:**

This integrated workflow combining MiCAPs, ePCR, and PSR demonstrates a practical approach for marker development and diversity assessment in polyploid species lacking reference genomes. Despite the genetic complexities inherent in Zingiberaceae, especially potential polyploidy, our approach proved highly effective in establishing a robust phylogenetic framework and enabling comprehensive genetic diversity assessment. The novel set of 612 SSR marker candidates represents a significant resource that will facilitate future genetic studies focused on the diversity, evolutionary relationships, conservation, and sustainable utilization of valuable Zingiberaceae species

## 1 Introduction

Zingiberaceae, one of the most widely distributed plant families in tropical and subtropical regions, comprises *ca*. 50 genera and over 1,600 species. It includes a variety of species that are renowned for their pharmacological properties and traditional culinary uses (Hartati et al., 2014; Saha et al., 2020). The classification of this family was first proposed in 1889 and refined through time. The current accepted classification includes four tribes (Globbeae, Hedychieae, Alpinieae, and Zingibereae) (Kress et al., 2002). Earlier classification methods were mostly based on morphological characteristics. Later, molecular markers such as the internal transcribed spacer (ITS) and plastid *mat*K regions (Kress et al., 2002; Theerakulpisut et al., 2012), chloroplast genomes (Li et al., 2019; Liang et al., 2020; Jiang et al., 2023; Xia et al., 2024), and both nrDNA and cpDNA (Ngamriabsakul et al., 2004) were introduced. These studies have provided molecular evidence for taxonomy.

Our research focuses on several species within the Zingibereae tribe, which possess value in various aspects. Ginger (*Zingiber officinale* Roscoe) is a widely used spice and traditional herbal medicine worldwide. Evidence from *in vitro* studies, animal experiments, and epidemiological research suggests that ginger and its extracts have inhibitory effects against a variety of diseases (Srinivasan, 2014; Prasad and Tyagi, 2015). Myoga (*Zingiber mioga* (Thunb.) Roscoe) originated in Japan and cultivated for its edible flower buds and exhibits several biological activities (Stirling, 2004; Abe et al., 2006; Chan et al., 2023). *Zingiber barbatum* Wall. is an underutilized medicinal species with anti-inflammatory and analgesic properties (Wicaksana et al., 2011; Wicaksana, 2012; Aung, 2016). *Curcuma* species, including *Curcuma aromatica* Salisb., *Curcuma longa* L., *Curcuma zedoaria* (Christm.) Roscoe, and *Curcuma amada* Roxb., are rich sources of essential oil, also widely used as food additives and for various medicinal purposes (Lobo et al., 2009; Al-Reza et al., 2010; Albaqami et al., 2022). Especially turmeric (*C. longa*) and its extract exhibit anti-inflammatory, anti-human immunodeficiency virus, anti-bacteria, antioxidant effects and nematocidal activities (Araujo and Leon, 2001; Ammon and Wahl, 2007). Essential oils extracted from *Hedychium* J. Koenig species possess anti-microbial, anti-oxidant, and anti-inflammatory properties, and some species are also cultivated as ornamental plants (Joshi et al., 2008; Van Kiem et al., 2011; Hartati et al., 2014). *Kaempferia parviflora* Wall. ex Baker and *K. galanga* L. are used as food ingredients, traditional medicine, and proved to possess anti-obesity, anti-inflammatory, anti-allergenic, and anti-cancer properties (Tewtrakul et al., 2009; Kobayashi et al., 2015; Yoshino et al., 2018; Kumar, 2020; Khairullah et al., 2021). *Rhynchanthus longiflorus* Hook.f. is an endangered species which is rarely reported (Prasanthkumar et al., 2005; Debnath and Vijayan, 2024).

Among these species, some are considered under-utilized, and the lack of basic molecular biological information severely hampers their conservation and utilization. Simple Sequence Repeat (SSR, also known as microsatellite) markers are particularly suitable for under-utilized species because they do not require a complete reference genome and can still provide relatively high levels of polymorphic information in even closely related samples (Grover and Sharma, 2016; Foster et al., 2020). This is especially important for Zingiberaceae species since the genome is large and complicated (Cheng et al., 2021; Li et al., 2021). Traditional SSR marker development based on Sanger sequencing is both costly and time-consuming. Although the advent of next-generation sequencing (NGS) has significantly reduced the cost, it remains challenging for under-utilized species to secure sufficient funding for whole-genome sequencing research (Senan et al., 2014; Ramesh et al., 2020). Microsatellite capture sequencing (MiCAPs) offers a more efficient alternative for SSR detection by enriching the DNA library with SSR probes. This method requires only about 1% of the whole genome to generate sufficient SSR and flanking sequence data for marker development (Tanaka et al., 2018; Shi et al., 2024). Currently, species such as *Z. mioga*, *Z. barbatum*, and *R. longiflorus* lack available SSR information. Moreover, SSR markers can sometimes be unsuitable for cross-species application, further limiting their utility (Ramesh et al., 2020). With the significantly reduced cost using MiCAPs, sequencing multiple species and evaluating markers’ transferability become more possible.

Given these challenges and opportunities, the primary objectives of this study are: (i) to develop a comprehensive set of polymorphic SSR markers for under-utilized Zingiberaceae species where such resources are currently lacking; (ii) to assess the transferability of these markers across closely related species within the family; and (iii) to investigate the genetic diversity patterns and phylogenetic relationships among major genera using SSR-enriched genomic data. To address these objectives, we applied the MiCAPs approach to a total of 148 accessions representing 14 species within the Zingiberaceae family. Using the resulting data, we constructed a phylogenetic tree and developed a large set of SSR marker candidates. These efforts not only provide valuable molecular resources for future genetic and evolutionary studies but also contribute to the conservation and sustainable use of under-utilized Zingiberaceae species.

To achieve these objectives, we employed an integrated workflow combining MiCAPs for SSR enrichment, followed by bioinformatic analyses for marker development and diversity assessment, as detailed below.

## 2 Materials and methods

### 2.1 Plant materials

In the current study, 160 accessions were collected and sequenced using the MiCAPs method. Among them, 148 accessions were from 14 Zingiberaceae species, and an additional 12 Musaceae accessions were selected as outgroups. Detailed information of all the accessions, including species name and passport data can be found in **Supplementary Table S1**. Species nomenclature follows Plants of the World Online (POWO, http://www.plantsoftheworldonline.org/, accessed on 26 October 2025) facilitated by the Royal Botanic Gardens, Kew. The rhizome samples were obtained through SMTA (Standard Material Transfer Agreement) of FAO ITPGRFA and/or individual MTAs (Material Transfer Agreement) with the owners through the corresponding authorities.

Plants were grown in Gene Research Center, University of Tsukuba, Japan under controlled greenhouse condition. Fresh young leaf samples were collected and kept at -80°C till DNA extraction.

### 2.2 DNA extraction, library establishment and sequencing

The detailed experimental procedures, including library construction, SSR enrichment, and sequencing, were performed as described in our previous work (Shi et al., 2024), which followed the MiCAPs protocol developed by Tanaka et al. (2018) with minor modifications. A brief overview of the workflow is provided below; readers are referred to the original MiCAPs publication (Tanaka et al., 2018) for comprehensive methodological details, including the design of biotinylated SSR capture probes.

Genomic DNA was extracted from the frozen young leaf tissue using a modified CTAB protocol (Doyle and Doyle, 1987) supplemented with 0.02g polyvinylpyrrolidone (PVP) per sample. DNA quality and integrity were evaluated by 2% agarose gel electrophoresis and NanoDrop 2000c spectrophotometer (Thermo Fisher, Waltham, MA, USA).

Subsequently, 100 ng of DNA was digested with EcoRI-HF (New England Biolabs, Ipswich, MA, USA) and HindIII-HF (New England Biolabs, Ipswich, MA, USA), ligated to custom adapters, and size-selected using AMPure XP magnetic beads (Beckman Coulter, Bera, CA, USA), following the Flexible ddRAD-seq framework (Ando et al., 2018). The size-selected fragments were amplified with dual-index primers through high-fidelity PCR.

For SSR enrichment, the pooled libraries were hybridized with a biotinylated (GA)_10_ probe and captured using Dynabeads MyOne streptavidin C1 beads (Life Technologies, Carlsbad, CA, USA). It should be noted that this probe specifically captures SSR-containing fragments with GA/AG/CT/TC motifs, which limits the diversity of marker types recovered and may introduce bias in SSR density estimates across different genomic regions or species. The enriched libraries were further amplified, purified, and assessed for quality and concentration. Sequencing was performed on an Illumina MiSeq platform (Illumina, San Diego, CA, USA) to generate 2 × 300 bp paired-end reads.

### 2.3 Raw data cleaning, clustering, and phylogenetic analysis

Raw sequencing reads were initially processed using fastp v0.23.2 (Chen et al., 2018) with default parameters to remove low-quality sequences and adapters. The resulting clean paired-end reads were merged using FLASh v1.2.11 (Magoc and Salzberg, 2011) with a maximum overlap of 500 bp and a minimum overlap of 60 bp.

Subsequently, similar merged reads were clustered using CD-HIT-EST v4.6 (Li and Godzik, 2006) to reduce redundancy. SSR polymorphism was inferred based on sequence redundancy, using common sequences containing SSR motifs as reference sequences.

For polymorphism detection and phylogenetic analysis, we followed the MiCAPs protocol (Tanaka et al., 2018). Briefly, sequence data from all samples were combined and re-clustered using CD-HIT-EST to establish a reference sequence set. Individual sample reads were mapped to the reference using CLC Genomics Workbench v9.5 (Qiagen, Denmark), and consensus sequences for each sample were generated. While this consensus-based approach may collapse allelic variation in polyploid accessions, it provides a practical framework for marker development and genus-level diversity assessment without requiring specialized polyploid-aware algorithms.

SSR loci were identified from the consensus sequences using SSRIT (Temnykh et al., 2001), and polymorphism data were extracted to construct a genotype matrix. Genetic distances among samples were calculated using the distance matrix method implemented in Populations v1.2.30 (Langella, 1999). A phylogenetic tree was then constructed based on the distance matrix using the neighbor-joining method in MEGA7 (Kumar et al., 2016). To assess the robustness of the phylogenetic relationships, bootstrap analysis was performed with 1,000 replicates. The final tree was visualized in a circular layout to facilitate the interpretation of relationships among the 160 accessions and to clearly display the major phylogenetic clades within Zingiberaceae.

### 2.4 SSR detection, primer design and candidate marker *in silico* evaluation

Clustered SSR-containing reads were searched using MISA (Thiel et al., 2003; Beier et al., 2017) (MIcroSAtellite identification tool) v2.1. Definitions of unit size and min repeats were 2-6, 3-5, 4-5, 5-5, and 6-5; max interruption was 100 bp. Then, only perfect SSRs with a size between 30 and 70 nt were selected with a custom python script. Considering that library is established from AG-motif probe enriched fragments, only those SSRs containing AG, GA, TC, and CT were included.

Primer3 v.0.4.0 (Koressaar and Remm, 2007; Untergasser et al., 2012) was employed to design five primer sets for each SSR locus. These marker candidates were deduplicated with similar threshold 90% by a custom script.

*In silico* evaluation of these marker candidates was then conducted with ePCR (Schuler, 1997). The sts size ranged from 100 to 1000, word size set as 12, margin 3000, no mismatches or indels allowed.

### 2.5 Diversity analysis based on polymorphic ssr retrieval

After the establishment of a phylogenetic tree, species under *Zingiber* and *Curcuma* were given focus for a diversity analysis. The list of plants in these groups can be found in **Supplementary Table S1**.

Polymorphic SSR Retrieval (PSR) (Cantarella and D’Agostino, 2015) is a Perl package for identifying polymorphic SSRs from NGS data and providing quantitative information of each SSR locus for each call. For PSR analysis, individual sample reads were mapped to the reference sequences using Bowtie2 v2.3.5 (Langmead and Salzberg, 2012), generating BAM (Binary Alignment Map) files that contain alignment information for each read. These BAM files, together with SSR annotations from MISA output (described above), were then processed by PSR with minimum read depth set to 3 (-t 3) and default parameters for variant calling (-p) to extract SSR genotype data, including allele sizes and copy numbers at each polymorphic locus. Principal coordinate analysis (PCoA) based on genetic distance was processed with Microsoft 365^®^ Excel^®^ plug-in GeneAlEx 6.503 (Peakall and Smouse, 2012; Smouse et al., 2017). From PSR output, loci present in at least 20% of the accessions were filtered for heatmap visualization. Presence/Absence heatmap of locus in each accession is drawn by a custom python script: if one locus is present in PSR output, it is marked as black, otherwise white.

To assess the distribution and transferability of SSR loci across species within each genus, presence/absence heatmaps were generated using a custom Python script. In these heatmaps, each SSR locus that passed the 20% frequency threshold is represented as a column, and each accession is represented as a row. A cell is colored black if the SSR locus is present in that accession and white if absent. To reveal the structural relationships among SSR loci and identify groups of loci with similar distribution patterns, hierarchical clustering analysis was performed on the SSR loci. Jaccard distance, which is particularly suitable for binary presence/absence data, was calculated between all pairs of loci using the scipy.spatial.distance.pdist function (Virtanen et al., 2020). Hierarchical clustering was then conducted using the average linkage method (scipy.cluster.hierarchy.linkage), which groups loci based on the average pairwise distances between members of different clusters.

## 3 Results

### 3.1 Overview of sequencing results

A total of 160 accessions, including 148 from Zingiberaceae and 12 from Musaceae, were subjected to MiCAPs. In total, 21.78 million raw reads were generated, corresponding to approximately 5.31 Gb of sequence data. On average, each accession yielded 136,124 reads and 33.19 Mb of raw data, with a Q30 base percentage of 91.70% (**Table 1**). After quality filtering using fastp, the proportion of Q30 bases increased to 93.46%, indicating good sequencing quality. The accession with the fewest sequencing bases was Z0106 (*C. amada*), with 12.41 Mb of bases, whereas the accession with the highest number of sequencing bases was MS3–1 from Musaceae (outgroup), with 64.77 Mb. The mean value of clean reads for each accession was 21.23 Mb (**Supplementary Tables S2**-**S8**).

**Table 1:** Statistics of MiCAPs data.

Category	Reads	Bases	Q30 bases
Average (Before)	136,124	33.19 M	91.70%
All (Before)	21.78 M	5.31 G	N/A
Average (After)	132,679	31.68 M	93.46%
All (After)	21.23 M	5.07 G	N/A

**Figure 1:**
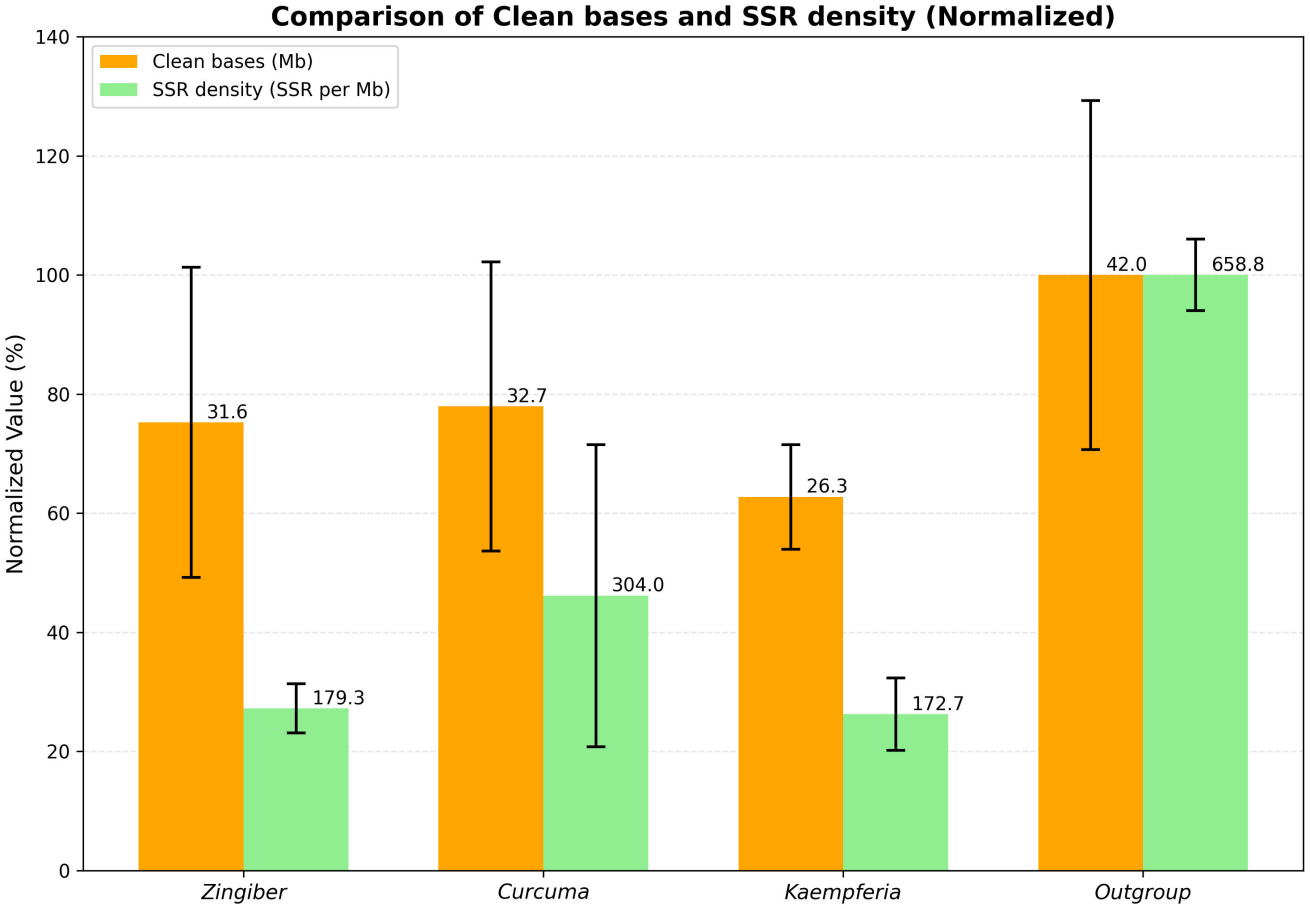
Comparison of clean bases and SSR density across major plant groups. Clean bases and SSR density were compared among three genera (*Zingiber*, *Curcuma*, and *Kaempferia*) and the outgroup Musaceae. Within each dataset, the values were normalized by setting the highest value (Musaceae) to 100%, with other values scaled accordingly.

Among the three most represented genera (*Zingiber*, *Curcuma*, and *Kaempferia*), the clean bases showed relatively minor differences, with *Zingiber* and *Curcuma* being particularly close in their data yield (**Figure 1**).

### 3.2 SSR mining and candidate marker development

Clustered sequences were used for SSR mining. In total, 10,414,651 sequences covering approximately 2.69 Gb of sequence data were examined, leading to the identification of 769,841 SSRs. The average SSR density was calculated to be 274.63 SSRs per Mb of sequence.

The SSR density across different groups showed substantial variation, ranging from a minimum of 108.77–188 to a maximum of 845.22 SSRs per Mb.

**Figure 2:**
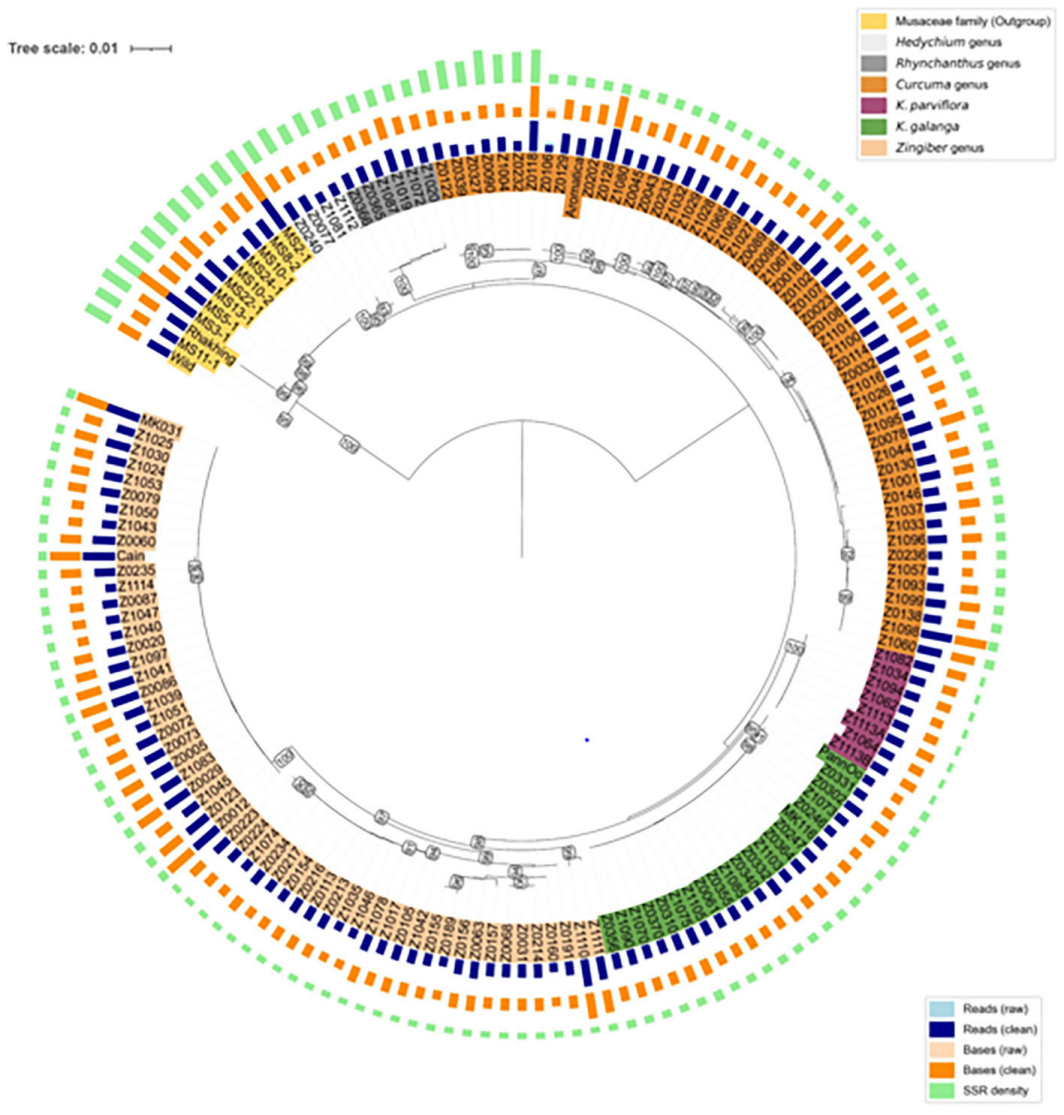
Circular phylogenetic tree of 160 accessions based on SSR-enriched sequences. Branch length represents genetic distance. Bootstrap values are shown as numbers. Four concentric rings are displayed from inside to outside: accession numbers, number of reads, sequence bases, and SSR density. The color bands for accession numbers (innermost) represent the biological classification of each accession, including Outgroup Musaceae family, *Hedychium* genus, *Rhynchanthus* genus, *Curcuma* genus, *K*. *parviflora*, *K*. *galanga*, and *Zingiber* genus.

As shown in **Figure 1** and **Figure 2**, the SSR density differed markedly among the four groups (*Zingiber*, *Curcuma*, *Kaempferia*, and Musaceae), with one *Curcuma* branch exhibiting notably higher SSR enrichment relative to *Zingiber*, *Kaempferia*, and other *Curcuma* accessions.

Further SSR marker development was conducted at the species level (**Table 2**). The number of SSR loci identified per accession varied across species, with *R. longiflorus* yielding the highest average (7259 SSRs per accession) and *Z. barbatum* the lowest (2180 SSRs per accession).

**Table 2:** SSR marker candidate statistics of each species.

Species	Number of accessions	Average SSR loci per accession	SSR marker candidates	Highly polymorphic markers (≥5 alleles)
*C. amada*	18	6,018	11,566	130
*C. aromatica*	16	4,629	10,016	119
*C. longa*	10	3,864	6,507	91
*K. galanga*	21	2,620	7,129	68
*R. longiflorus*	6	7,259	5,167	63
*Z. barbatum*	26	2,180	5,775	65
*Z. officinale*	20	4,048	12,411	76

The number of SSR marker candidates designed for ePCR screening ranged from 5167 in *R. longiflorus* to 12,411 in *Z. officinale*. Notably, a considerable number of markers with high allelic diversity (defined as ≥5 alleles) were identified via ePCR, ranging from 63 in *R. longiflorus* to 130 in *C. amada*. Data for *K. parviflora* is excluded due to prior publication. The developed SSR marker candidates can be found in **Supplementary Tables S2**-**S8**.

### 3.3 Phylogenetic tree construction based on SSR-enriched sequences

To investigate the genetic relationships among the 160 accessions, a circular phylogenetic tree was constructed based on consensus sequences (**Figure 2**).

The Musaceae accessions (yellow) formed a well-supported and isolated clade (bootstrap support = 100%), serving as an outgroup relative to the Zingiberaceae species. Within Zingiberaceae, the accessions were further divided into two major clades, designated as Clade I and Clade II.

Clade I is primarily comprised of the accessions from *Rhynchanthus* (95%), *Hedychium* (100%), and *Curcuma* (97%), indicating strong internal consistency within these genera. Clade II mainly included accessions of *K. parviflora* (100%), *K. galanga* (95%), and *Zingiber* (98%), also demonstrating high bootstrap support values and clear genetic separation among these groups.

### 3.4 Genetic diversity on major subclades using polymorphic SSR retrieval

We continue to focus on two major subclades representing *Curcuma* and *Zingiber* genus.

**Figure 3:**
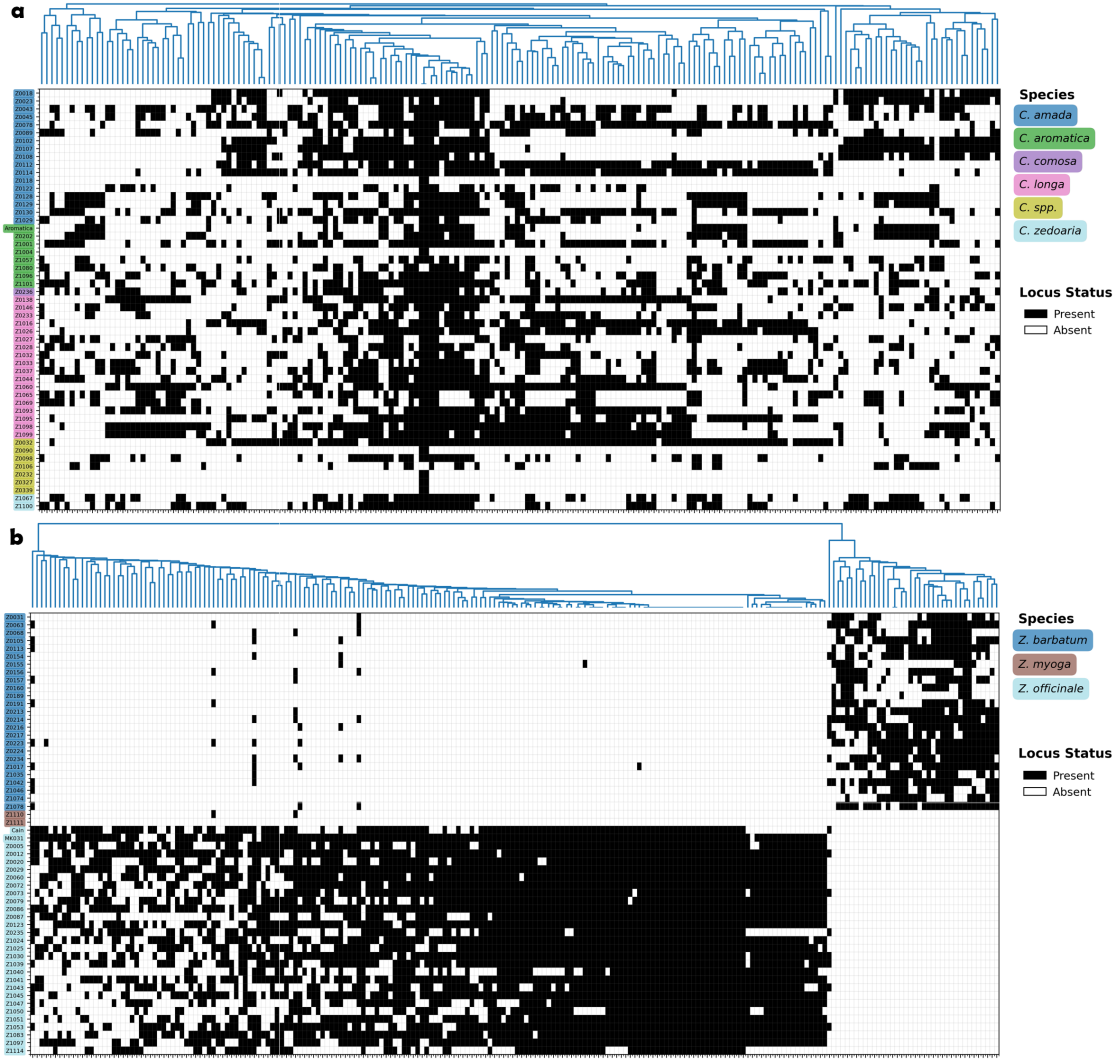
Presence/absence heatmap of filtered loci per accession. Loci present in at least 20% of the accessions are filtered for heatmap visualization. Black indicates presence, white indicates absence. **(a)***Curcuma* species. **(b)***Zingiber* species.

As shown in **Figure 3**, certain SSR loci exhibit transferability within *Curcuma* species, appearing simultaneously across multiple species. A total of six SSR loci were present in more than 75% of *Curcuma* accessions, whereas this number was zero for *Zingiber*. However, from another perspective, *Curcuma* species proved difficult to differentiate using SSR markers, while the three *Zingiber* species were distinctly separated from each other. This contrasting pattern suggests differential utility of SSR markers across these related genera within Zingiberaceae.

**Figure 4:**
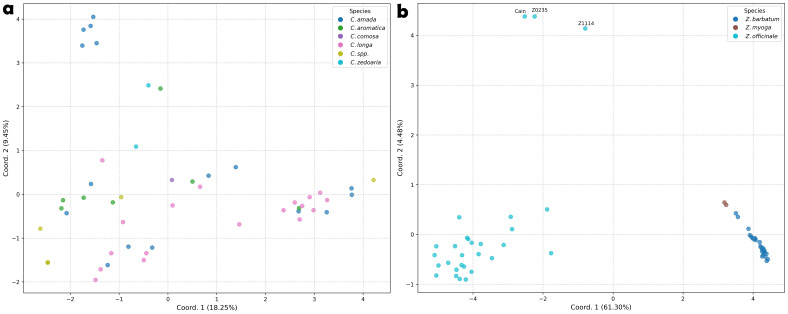
Principle Coordinate Analysis of *Curcuma* and *Zingiber* species based on Nei’s Genetic Distance calculated from PSR detected SSR loci. **(a)***Curcuma* species. **(b)***Zingiber* species.

The PCoA plots based on genetic distances corroborated the heatmap and tree-based clustering findings, revealing distinct clustering patterns between genera (**Figure 4**). *Curcuma* accessions exhibited considerable admixture with no clear clustering pattern, with the first two axes explaining only a modest proportion (27.70%) of the total genetic variation. In contrast, *Zingiber* accessions formed three well-defined clusters that aligned precisely with their taxonomic classification. Notably, three *Z. officinale* accessions—Cain, Z0235, and Z1114—were positioned at considerable distance from the main cluster.

To further characterize the genetic diversity differences between the two genera, population genetic diversity indices were calculated (**Table 3**). *Curcuma* exhibited notably higher genetic diversity across multiple indices compared to *Zingiber*. Specifically, *Curcuma* showed higher values for the number of alleles (Na = 1.268 vs. 0.519), number of effective alleles (Ne = 1.114 vs. 0.414), Shannon’s information index (I = 0.238 vs. 0.059), and expected heterozygosity (He = 0.148 vs. 0.035). These quantitative measures corroborate the PCoA results and support the interpretation that *Curcuma* possesses greater genetic complexity, while *Zingiber* maintains a more conserved genetic background. The higher fixation index in *Curcuma* (F = 0.621) compared to *Zingiber* (F = 0.467) further suggests stronger population differentiation within *Curcuma*, consistent with its allopolyploid nature and history of hybridization.

**Table 3:** Genetic diversity indices for *Curcuma* and *Zingiber* populations.

Index	*Curcuma*		*Zingiber*	
	Mean	SE	Mean	SE
Na (No. of different alleles)	1.268	0.032	0.519	0.032
Ne (No. of effective alleles)	1.114	0.025	0.414	0.023
I (Shannon’s Index)	0.238	0.011	0.059	0.007
Ho (Observed heterozygosity)	0.053	0.005	0.019	0.003
He (Expected heterozygosity)	0.148	0.007	0.035	0.004
uHe (Unbiased He)	0.172	0.008	0.036	0.005
F (Fixation index)	0.621	0.017	0.467	0.018

Having established these patterns of SSR distribution, marker diversity, and genetic relationships among Zingiberaceae species, we now discuss the biological and methodological implications of these findings.

## 4 Discussion

### 4.1 SSR statistics and SSR marker candidates

In the current study, we located 769,841 SSRs with the average SSR density 274.63 SSRs per Mb of sequence. The SSR density was similar to previous study reported as 216.1–367.3 SSRs/Mb in *Curcuma* (Ye et al., 2024).

In our previous study, we developed and validated SSR markers for *K. parviflora* using the MiCAPs workflow, demonstrating its reliability (Shi et al., 2024). In the current study, we provided SSR marker candidates—validated through ePCR and PSR—for a range of Zingiberaceae species that previously lacked such resources, enabling potential future studies and applications.

The SSR-containing fragments captured and sequenced by MiCAPs account for only about 1% of the whole genome (Shi et al., 2024), significantly reducing the overall cost. This is particularly advantageous for under-utilized species. For well-studied species, the reduced cost makes it feasible to sequence multiple accessions, allowing for a more comprehensive understanding of genetic variation and enabling the assessment of SSR marker transferability across closely related species.

For example, we identified six SSR loci in *Curcuma* that are shared among multiple *Curcuma* species, suggesting a degree of cross-species applicability. However, this is not always the case—among the three *Zingiber* species, most loci were found to be species-specific, indicating limited transferability. On the other hand, this species specificity offers a potential advantage for rapid species identification. These differential patterns of SSR distribution suggest distinct applications for the developed markers: the species-specific SSR loci identified in *Zingiber* are particularly suitable for germplasm authentication and species identification in breeding programs and conservation efforts, while the transferable SSR loci observed across multiple *Curcuma* species offer potential for comparative genetic studies and cross-species applications within this genus, which is valuable given the prevalence of hybridization and allopolyploidy in *Curcuma*.

At the intra-species level, when screening for SSR marker candidates, we selected perfect SSRs with repeat lengths ranging from 30 to 70 nucleotides. This size range is often associated with higher levels of polymorphism, making these SSRs particularly valuable for genetic studies (Weber, 1990; Xu et al., 2000; Temnykh et al., 2001; Patil et al., 2021). The rationale for this selection is twofold: first, SSRs of this length tend to exhibit greater allelic diversity due to higher mutation rates, facilitating the detection of fine-scale genetic differences within populations; second, they remain sufficiently short to ensure robust and reproducible PCR amplification under standard laboratory conditions. In some studies, such SSRs are also referred to as Class I (Patil et al., 2021) or HvSSR (highly variable SSR) (Singh et al., 2010), highlighting their potential for revealing significant intra-species genetic variation. These highly polymorphic SSR markers (developed from 30–70 nt perfect SSRs) therefore provide robust tools for population genetic analysis and diversity assessment in Zingiberaceae species.

The choice of MiCAPs over alternative enrichment approaches merits consideration in the context of recent phylogenomic studies in Zingiberaceae. While target capture (hybrid capture) methods have been successfully applied to obtain chloroplast genomes for phylogenetic reconstruction in this family (Li et al., 2019; Xia et al., 2024), these approaches primarily recover organellar sequences suitable for deep phylogenetic inference at family or tribe levels (Pezzini et al., 2023). In contrast, MiCAPs specifically enriches nuclear genomic regions containing SSRs, which exhibit higher mutation rates and therefore greater polymorphism levels compared to chloroplast markers (Li et al., 2020). This fundamental difference in target loci makes MiCAPs particularly advantageous for population-level studies and diversity assessment rather than deep phylogenetic reconstruction. Furthermore, the cost structure differs between approaches: while target capture requires initial investment in custom bait design, the per-sample sequencing costs can be reduced when applied to large sample sets (Pezzini et al., 2023). However, for under-utilized species with limited prior genomic resources—such as several species examined in this study—MiCAPs offers a more accessible entry point, requiring only approximately 1% genome coverage (Shi et al., 2024) to generate sufficient polymorphic markers without the need for reference genome-based bait design. These complementary methodologies serve distinct research objectives: chloroplast-based approaches excel at resolving deep evolutionary relationships, whereas nuclear SSR enrichment via MiCAPs provides the high-resolution markers necessary for intraspecific genetic diversity analysis and germplasm characterization.

### 4.2 Phylogenetic and diversity study of Zingiberaceae

In our phylogenetic tree (**Figure 2**), *Hedychium*, *Rhynchanthus*, and *Curcuma* are in the same cluster, while *Kaempferia* and *Zingiber* are in another. This genus-level phylogenetic relationship is concordant with previous studies based on chloroplast DNA (Cui et al., 2019; Li et al., 2019; Li et al., 2021), internal transcribed spacer (ITS) regions, and *matK* (Kress et al., 2002) sequences. While these traditional phylogenetic markers (cpDNA, ITS, *matK*) have proven effective for resolving higher-level taxonomic relationships, nuclear SSR markers offer complementary advantages for fine-scale diversity analysis due to their higher polymorphism levels and biparental inheritance (Kress et al., 2002; Li et al., 2020). However, we acknowledge that our neighbor-joining approach has inherent limitations, including inability to model site heterogeneity and susceptibility to long-branch attraction (Gascuel and Steel, 2006). Moreover, SSRs are biparentally inherited and multi-allelic, differing from the uniparentally inherited cpDNA commonly used in phylogenetic studies (Ellegren, 2004). We acknowledge that formal topology tests (e.g., Shimodaira-Hasegawa test) were not performed, as the primary objective of this study was a comprehensive SSR study rather than rigorous phylogenetic reconstruction. Therefore, at finer taxonomic scales—particularly within genera where hybridization and polyploidy are prevalent—our results are more appropriately interpreted as genetic similarity patterns rather than strict phylogenetic relationships. Nonetheless, the broad congruence at the genus level, supported by high bootstrap values (95–100% for major genera), suggests that SSR-based analysis can effectively capture major evolutionary relationships among Zingiberaceae genera.

The PCoA provides further insights into the genetic relationships and diversity patterns among the *Curcuma* and *Zingiber* accessions (**Figure 4**). Notably, the *Curcuma* accessions exhibited a dispersed pattern without forming distinct clusters within the PCoA plot (**Figure 4a**). Correspondingly, the first two principal coordinate axes explained only 27.70% of the total observed genetic variation for this genus. This lack of clear grouping and the low explanatory power of the initial axes underscore the substantial genetic complexity within *Curcuma* (Záveská et al., 2016; Skopalíková et al., 2023), aligning with our observation of SSR loci distribution (**Figure 3a**).

In striking contrast, the *Zingiber* accessions demonstrate a much clearer genetic structure, dividing into two well-defined major groups (**Figure 4b**). One cluster exclusively comprises all accessions identified as *Z. barbatum* and *Z. mioga*, while the other includes all the *Z. officinale* (ginger) accessions. This distinct separation suggests a more conserved genetic background or stronger reproductive barriers between these *Zingiber* groups compared to the pattern observed in *Curcuma*. Quantitative genetic diversity indices further support this interpretation (**Table 3**): *Zingiber* exhibited substantially lower values for key diversity measures including Shannon’s index (I = 0.059 vs. 0.238), expected heterozygosity (He = 0.035 vs. 0.148), and number of alleles (Na = 0.519 vs. 1.268) compared to *Curcuma*, corroborating the observed differences in genetic structure and complexity between the two genera.

Within these *Zingiber* clusters, the analysis also revealed differences in genetic diversity; the *Z. officinale* cluster displayed a wider dispersion, indicating greater genetic variation among the ginger accessions, whereas the *Z. barbatum* accessions showed lower genetic diversity within this group. Furthermore, attention was drawn to three specific ginger accessions (‘Cain’, ‘Z0235’, and ‘Z1114’) that were positioned at a considerable distance from both major *Zingiber* clusters, suggesting significant genetic divergence. This genetic distinctiveness could potentially indicate unique germplasm resources within *Z. officinale* and warrants further investigation in future breeding programs and evolutionary studies of the Zingiberaceae family.

It is important to clarify that our methodology is fundamentally based on SSR repeat number polymorphism rather than SSR-flanking sequence analysis (Tanaka et al., 2018). While this avoids complications from potentially paralogous flanking sequences, SSRs are inherently prone to homoplasy and size homoplasy (Estoup et al., 2002). However, the large number of SSR loci analyzed in this study (hundreds of polymorphic loci across the dataset) likely compensates for potential homoplasious evolution at individual loci, as the high overall variability enables robust inference of genetic relationships despite occasional convergent mutations (Estoup et al., 2002). Regarding polyploidy, our workflow simplifies complexity to facilitate marker development: consensus sequence construction may collapse allelic variation in polyploids, and PSR was applied with default diploid-like settings (Cantarella and D’Agostino, 2015). Although this simplified approach may not capture full allelic complexity, it proved effective for generating actionable SSR candidates and revealing diversity patterns without requiring specialized polyploid-aware algorithms (Rothfels, 2021). Additionally, the use of a (GA)_10_ biotinylated probe for SSR enrichment may introduce capture bias toward genomic regions or subgenomes enriched in GA/AG/CT/TC-type repeats, potentially affecting SSR recovery efficiency across different species or homeologous chromosomes in polyploid accessions. This bias should be considered when interpreting interspecific differences in SSR density.

### 4.3 Hybridization characteristics and SSR loci distribution

Each species has different genome size, and some of the accessions are polyploid within a given species classification. The chromosome number, ploidy level and genome size (2-c value or genome assembly) of some major species we used in current study that were already reported are: *C. longa* (2n=63; 3x or 4x; 2.60–2.85 pg) (Leong- Škorničková et al., 2007; Yin et al., 2022; Aswathi and Prasath, 2024), *C. amada* (2n=42; 6x; 1.82–1.88 pg) (Leong- Škorničková et al., 2007), *C. aromatica* (2n=42,63,86; 6x or 9x; 2.68–2.86 pg) (Leong- Škorničková et al., 2007; Shankar, 2021), *Z. officinale* (2n=22; 2x; 3.1Gb) (Cheng et al., 2021; Li et al., 2021), *Z. barbatum* (unknown; 2x, 3x or 4x; 2.90–5.98 pg) (Shukurova, 2016).

*Curcuma* plants display a great degree of diversity in ploidy levels with different chromosome numbers among different species (Ahmad, 2010; Chen et al., 2013; Lamo, 2017), and many of them are of allopolyploid in origin, which are therefore hybrids (Záveská et al., 2016). Hybridization within *Curcuma* is common in nature and is also applied in the horticultural industry (Anuntalabhochai et al., 2007; Záveská et al., 2016). This may help explain why the SSR loci in *Curcuma* are often shared across multiple species. Moreover, even different accessions of the same species may possess entirely distinct SSR loci (**Figure 3a**), a phenomenon that could be attributed to their allopolyploid nature.

Interestingly, we observed a particular branch within *Curcuma* (Z0122, Z0339, Z0327, Z0090, Z1004, Z0232, and Z0118) that exhibits a notably higher SSR density (**Figure 2**), and in heatmap they don’t share many SSR loci with other *Curcuma* species (**Figure 3a**). However, background information about this group remains limited; we were only able to identify two accessions within the cluster as *C. amada* and one as *C. aromatica* (**Supplementary Table S1**). The unusually high SSR density and unique SSR loci in this lineage may be related to their ploidy level and evolutionary history. Alternatively, as discussed earlier regarding probe capture bias, this elevated SSR density could simply reflect higher capture efficiency of GA-type repeats by the (GA)_10_ biotinylated probe in this particular lineage, rather than true genomic differences. Further investigation using flow cytometry (FCM) or whole genome sequencing will be necessary to determine exact ploidy and genetic makeup, and to distinguish between genuine genomic expansion and methodological artifacts.

In contrast, this pattern is less evident in *Zingiber*. Although *Z. barbatum* accessions used in current study are validated to be in triploid and tetraploid forms (Shukurova, 2016), neither its SSR density nor the distribution of SSR loci showed significant variation among different ploidy levels. This is also true with *K. galanga*, which has 4x and 5x forms (FCM data not published). This observation might be attributable to the strong reproductive isolation in these species.

In the case of *K. galanga*, PanOo was separated from the rest of the accessions having less SSR density. It could be attributed to its 4x ploidy level while the others are 5x (Lu et al., 2023).

## 5 Conclusion

In conclusion, the species investigated in this study primarily rely on vegetative propagation, and as the previous cytological studies indicated that some are regarded as allopolyploids, ordinary genetic markers could be challenging for systematic study. Compounding this, the genetic landscape is further complicated by considerable variation in genome size and the occurrence of intra-specific ploidy differences among accessions. Despite these complexities, our integrated approach employing SSR-enriched sequencing (MiCAPs) coupled with *in silico* SSR marker simulations (PSR) demonstrated remarkable effectiveness, particularly in efficiently generating actionable marker candidates from polyploid genomes without requiring specialized polyploid-aware algorithms. These methodologies successfully circumvented the obstacles posed by the diverse genetic complements, proving highly capable of establishing a robust phylogenetic framework across the Zingiberaceae family and facilitating a comprehensive evaluation of genetic diversity at the genus level. Furthermore, a significant outcome of this research is the development of a novel set of SSR marker candidates derived from the generated sequence data. These markers represent a valuable resource for species identification, population genetic analysis, and cross-species comparative studies, promising to significantly aid future genetic investigations focused on understanding the diversity, relationships, and conservation of under-utilized species within the Zingiberaceae family.

## Data Availability

The original contributions presented in the study are publicly available. This data can be found here: DDBJ database, accession numbers PRJDB18485 and PRJDB35866.
